# Neutralizing Antibodies Targeting the Conserved Stem Region of Influenza Hemagglutinin

**DOI:** 10.3390/vaccines8030382

**Published:** 2020-07-12

**Authors:** Sabari Nath Neerukonda, Russell Vassell, Carol D. Weiss

**Affiliations:** Center for Biologics Evaluation and Research, U.S. Food and Drug Administration, Silver Spring, MD 20993, USA; NagaVenka.Neerukonda@fda.hhs.gov (S.N.N.); Russell.Vassell@fda.hhs.gov (R.V.)

**Keywords:** influenza, hemagglutinin, broadly neutralizing antibodies, universal influenza vaccines

## Abstract

Influenza continues to be a public health threat despite the availability of annual vaccines. While vaccines are generally effective at inducing strain-specific immunity, they are sub-optimal or ineffective when drifted or novel pandemic strains arise due to sequence changes in the major surface glycoprotein hemagglutinin (HA). The discovery of a large number of antibodies targeting the highly conserved stem region of HAs that are capable of potently neutralizing a broad range of virus strains and subtypes suggests new ways to protect against influenza. The structural characterization of HA stem epitopes and broadly neutralizing antibody paratopes has enabled the design of novel proteins, mini-proteins, and peptides targeting the HA stem, thus providing a foundation for the design of new vaccines. In this narrative, we comprehensively review the current knowledge about stem-directed broadly neutralizing antibodies and the structural features contributing to virus neutralization.

## 1. Introduction

Influenza virus infection remains a constant threat to public health worldwide. Globally, annual seasonal influenza epidemics result in an estimated 3–5 million cases of severe illness and 0.25–0.5 million deaths with the occasional pandemics causing deaths in millions. Four types of influenza viruses, A, B, C, and D have been identified, of which only A, B, and C types cause illnesses in humans. Whereas both influenza A and B are responsible for annual seasonal epidemics, only influenza A is known to cause pandemics. Based on serological reactivity to two major surface proteins, hemagglutinin (HA) and neuraminidase (NA), influenza A viruses are subtyped into 18 HA and 11 NA subtypes, respectively [1,2]. The HA subtypes are classified based on structural similarity and antigenic phylogeny into two groups: group 1 (H1, H2, H5, H6, H8, H9, H11–H13, H16 and H17) and group 2 (H3, H4, H7, H10, H14 and H15). Avian reservoirs harbor a majority of the subtypes, but to date only H1, H2, H3, H5, H7 and H10 subtypes have been found in humans. Influenza B viruses are classified into two distinct phylogenetic lineages, Yamagata and Victoria [3].

**Figure 1:**
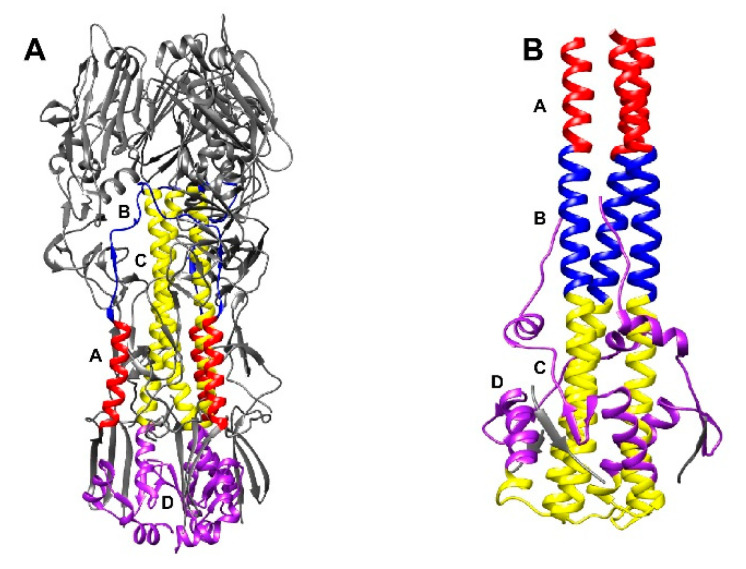
Hemagglutinin (HA) structures in the pre-fusion and post-fusion conformation. (**A**) Prefusion HA conformation with helices A (red), C (yellow), and D (purple) and B loop (blue) in each protomer colored distinctly (PDB code; 4FNK) [10]. (**B**) Post-fusion HA conformation colored as in (A) (PDB code; 1HTM) [11]. Note that in the post-fusion confirmation, the B loop folds into the helix B in the extended helix and most of HA1 is not present in this structure.

HA is synthesized as a monomer that assembles into a trimeric precursor (HA0), which upon cleavage by host proteases, becomes the fusion-competent mature trimer (HA) comprised of disulfide bond-linked HA1 and HA2 subunits (Figure 1A) [4]. Each HA trimer is a type I membrane glycoprotein composed of a membrane-distal globular head domain atop of the membrane-proximal stem domain. The receptor-binding site (RBS), present as a small depression on the head domain of HA1 subunit, mediates binding to the terminal sialosides on host cells, whereas the largely helical stem domain formed by the N- and C-terminal regions of HA1, and HA2 subunit facilitates membrane fusion [5]. During receptor-mediated endocytosis, the exposure to a low endosomal pH triggers the HA stem domain to undergo irreversible conformational changes leading to fusion between viral and cellular endosomal membranes (Figure 1B) [6].

HA glycans are required for proper protein folding, but they also contribute to immune evasion by masking antigenic epitopes or mimicking self-structures [7,8]. Influenza infection or vaccines induce neutralizing antibodies (nAbs) that are predominantly directed towards highly antigenic sites surrounding the RBS and typically interfere with binding to sialic acid receptors on target cells. RBS-directed nAbs inhibit the hemagglutination of red blood cells (HI activity), a surrogate measure of virus neutralization. However, these antigenic sites exhibit high plasticity generated by the error-prone viral polymerase, which facilitates antigenic variation (drift) due to immune selection [9]. As a result, most nAbs directed to these sites are strains specific and recognize only the matched or closely related virus strains [9]. In this regard, the mismatches between seasonal drifted strains and vaccine strains can lead to a loss in vaccine efficacy.

Occasionally, co-infection with more than one virus strain, typically in an animal host, can result in a re-assortment (or antigenic shift) of influenza genes from different viruses. Such an event can give rise to pandemic viruses bearing novel HA globular head domains against which humans have little to no herd immunity. For the above reasons, new vaccine strategies involving antigens that better focus immune responses to the highly conserved regions of HA are being developed [12]. Many advancements in the isolation of broadly nAbs (bnAbs) targeting the highly conserved regions of the HA head, including the antigenic sites of RBS (e.g., C139/1, S139/1, C05, 5J8, F045-092, F026-427, CH65), sites outside RBS (e.g., Fab6649, 441D6), vestigial esterase domain (e.g., H3v-47), and trimer interface (e.g., FluA-20) have been described thus far [10,13,14,15,16,17,18,19,20,21,22,23]. However, the neutralization breadth of a majority of head-directed antibodies is limited to a specific subtype.

The identification of a growing number of broadly nAbs that are directed to the more conserved stem domain of HA has catalyzed efforts to create new “universal” influenza vaccines that can protect against influenza strains from most subtypes that are potentially of seasonal, pandemic, or even zoonotic origin [24]. The neutralization breadth of stem-directed bnAbs (sbnAbs) range from multi-subtype to pan-group or even pan-flu (A and B type) viruses [25]. Many of these sbnAbs neutralize the virus by stabilizing HA in its prefusion conformation, preventing structural changes necessary for triggering fusion in acidified endosomes during viral entry [26,27]. In the HA2 subunit, the B-loop that bridges helices A and C in the prefusion state undergoes a loop to helix transition at a low-pH, resulting in an extended α-helix (C–B–A) (Figure 1). This transition brings the buried N-terminal fusion peptide in proximity to the endosomal membrane to trigger fusion. By inhibiting this transition, sbnAbs exhibits neutralization activity in virus microneutralization, pseudovirus neutralization, and plaque assays, in a manner that is independent of HI activity. Other sbnAbs inhibit the proteolytic cleavage of HA0 to HA, prevent particle egress [27,28,29], or inhibit NA activity by steric hindrance [30,31]. Furthermore, Fc-dependent effector functions of sbnAbs, including Ab-dependent cell cytotoxicity (ADCC), Ab-dependent cell phagocytosis (ADCP), and complement-dependent cytotoxicity (CDC) can also contribute to the elimination of virus and virus-infected cells [32]. In the present review, we give examples of sbnAb isolation approaches, mechanisms of antibody induction and neutralization, and protective efficacy in animal challenge models. We conclude our narrative with an overview of the vaccination strategies for eliciting sbnAbs and the remaining challenges that need to be overcome for the successful formulation of universal vaccines.

## 2. Stem-Directed bnAbs

### 2.1. Isolation Approaches of sbnAbs

**Figure 2:**
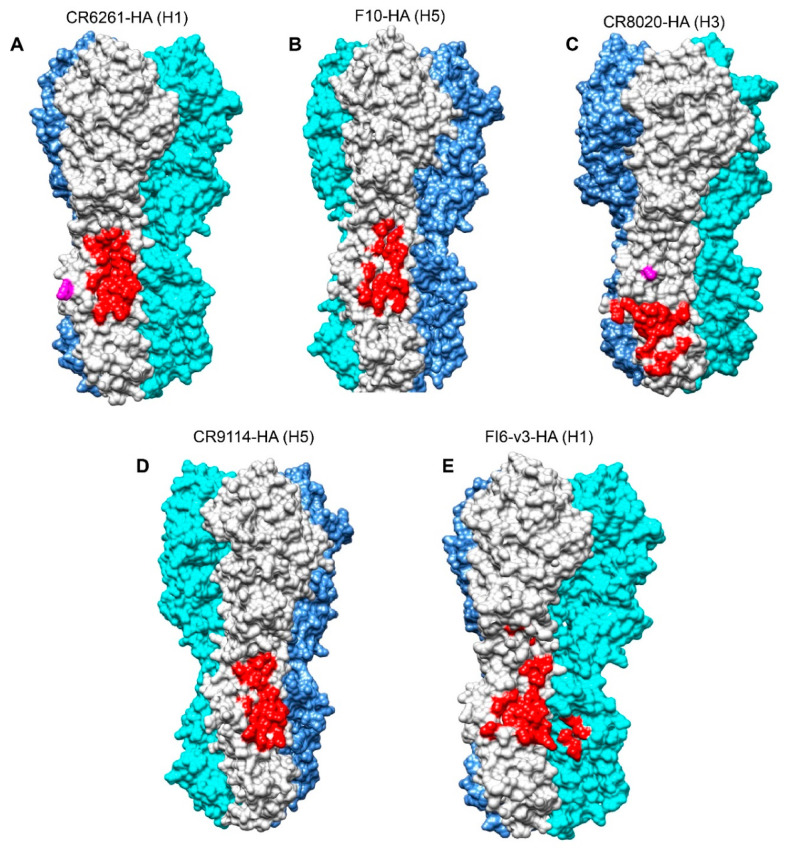
HA stem antibody epitope foot prints (in red). Each HA monomer is distinctly colored in light grey, cyan and cornflower blue (**A**) CR6261 epitope foot print on H1 HA (PDB code: 3LZG) [48]. (**B**) F10 epitope foot print on H5 HA (PDB code: 2FKO) [49]. (**C**) CR8020 epitope foot print on H3 HA (PDB code: 3ZTJ) [28]. (**D**) CR9114 epitope foot print on H5 HA (PDB code: 2FKO) [49]. (**E**) FI6v3 epitope foot print on H1 HA (PDB code; 3LZG) [48]. Note: Glycan (N21) that occludes the binding of CR8020 to group 1 HA in (A) or glycan (N38) reoriented for binding both group 1 and 2 HAs by CR9114 and FI6v3 in (B) is highlighted in magenta.

Several high throughput approaches have been used to isolate sbnAbs from mice, humans, and llamas. These include limiting the dilution cloning of peripheral blood mononuclear cells (PBMCs), Epstein Barr virus (EBV)-transformed/non-transformed human memory B-cells [33,34,35,36] and plasmablasts [37], and mouse spleen hybridomas [38,39]. Other approaches include phage display [14,27] and single-cell PCR cloning of heavy and light chain regions [40,41]. A majority of sbnAbs initially isolated in humans (e.g., CR6261, F10, and CR9114) utilized variable heavy chain (V_H_) 1–69 germline genes with stem interaction, primarily mediated by the heavy chain binding [26,41,42]. The conserved hydrophobic amino acid residues at the tip of the heavy chain complementary determining region (HCDR) 2 (I and F) and HCDR3 (Y) insert into the hydrophobic pockets of the HA stem to mediate the interaction [26]. These HCDR2 and HCDR3 residues together form a conserved IFY hydrophobic motif that is critical for the broadly neutralizing nature of these sbnAbs. Subsequently, the sbnAbs that use other germline families such as V_H_3–30, V_H_1–18, V_H_6-1, V_H_4–39, V_H_1–3 and V_H_3–23 have been identified [35,43,44,45,46]. Three sbnAbs of the V_H_3–30 germline family, FI6, 39.29 and 3I14, use both heavy and light chains to interact with the stem at a different angle of approach compared to the V_H_1–69 germline sbnAbs [28,37]. In further comparison, the V_H_3–30 germline sbnAbs use an extended HCDR3 to interact with the hydrophobic pockets that are occupied by the IFY motif of the V_H_1–69 germline sbnAbs [37]. Despite these differences, epitope footprints of the V_H_3–30 and V_H_1–69 germline sbnAbs are largely similar (Figure 2) [47]. Examples of various sbnAbs, their structural basis of neutralization, and structure-guided rational design of stem-directed mini-proteins and peptides are discussed in detail below. A more comprehensive list of the currently available sbnAbs and peptides targeting the HA stem of group 1, group 2, or both groups of viruses is presented in Table 1. Subtype-specific amino acid residue differences and mutations that confer resistance to stem antibody binding are listed in Table 2.

### 2.2. Group 1 HA Stem-Directed bnAbs and Small Proteins

#### 2.2.1. sbnAb C179

The sbnAb C179 was the first antibody reported to bind the HA stem and cross-neutralize multiple subtypes of Group 1 (H1, H2, H5, H6, H9) influenza A viruses [38]. C179 was originally isolated from the spleen hybridomas of mice twice immunized with the H2N2 virus. It uses both heavy and light chains for stem interaction. The C179 V_H_ chain binds to the HA stem using HCDR1 and HCDR3, that together form a linear cluster of hydrophobic residues resulting in a complementary hydrophobic interface that interacts with the hydrophobic groove in the HA stem surrounded by the fusion peptide, helix A, and the N-terminal segment of HA1 [51]. The light chain CDR1 (LCDR1) also forms hydrophobic interactions with the aliphatic portions of HA2. The C179 epitope spans the N- and C-terminal regions of HA1 (H3 numbered residues, 38, 40, 42, 291 to 293, and 318) and the N-terminal region of HA2 (residues 18 to 21, 38, 41 to 43, 45, 46, 52, and 56) including helix A. The C179 binds to an epitope similar to the epitope bound by group 1 sbnAbs, CR6261 and F10 (see below). A group-specific difference, T/A111 in group 2 HA2 instead of H111 in group 1 HA2, abrogates their binding to group 2 HAs by preventing favorable interactions with HCDR2 hot spot residues (F54 and F55) [51]. The earlier mapping of C179 resistance mutations identified two substitutions (HA1: T318K; HA2: V52E) in the H1 and H2 subtypes, respectively (Table 2) [38].

C179 exhibited potent in vitro neutralizing activity against seasonal H1N1 and H2N2, followed by H1N1pdm09, and to a much lesser extent, H5N1 viruses [50]. However, in mice challenge studies, the prophylactic administration of C179 (15 mg/kg) via the intranasal (i.n.) or intraperitoneal (i.p.) route led to the complete protection against challenge with seasonal H1N1, H1N1pdm09, and some H5N1 viruses [50]. The therapeutic administration of C179 was less effective against H1N1pdm09 and H5N1 viruses compared to the seasonal H1N1 viruses, although C179 extended the survival period of H1N1pdm09-infected mice. These studies highlighted the importance of other protection mechanisms besides neutralization, such as CDC, ADCC, and ADCP.

#### 2.2.2. sbnAb A06

The sbnAb A06 was obtained via the combinatorial phage library panned against H5N1 (A/Vietnam/1203/04) HA [53]. Phage libraries were constructed from the Turkish survivors of H5N1 (A/Turkey/65596/06) avian influenza virus infection. While the structural mapping of the A06 epitope on the HA stem is still awaited, the A06 epitope is predicted to encompass helix A. A06 uses the 1–69 V_H_ germ line region like remaining group 1 HA sbnAbs. A06 exhibited comparable in vitro neutralization potencies against seasonal H1N1, H1N1pdm09, and H5N1 viruses belonging to group 1, although earlier H1N1 (A/PR/8/34 and A/Texas/1991) strains required substantially higher concentrations of antibody to be effective [53]. Presumably, potential N-linked glycosylation sites proximal to the predicted epitope in A/PR/8/34 (amino acids 285–287) and A/Texas/1991 (amino acids 286–288) HA2 may sterically hinder antibody binding and neutralization. Furthermore, the prophylactic or therapeutic administration of A06 by i.p. route at 10 mg/kg led to the complete protection in mice challenged with mouse adapted (A/California/04/2009) or non-adapted (A/Netherlands/602/2009) H1N1pdm09 viruses [53].

#### 2.2.3. sbnAbs CR6261 and F10

CR6261 and F10 sbnAbs were isolated by panning combinatorial phage libraries constructed from seasonal influenza vaccinated individuals with the immobilized HA of H5N1 [41,52]. Both the bnAbs were derived from the V_H_1–69 germline and share a very similar breadth of heterosubtypic neutralization towards group 1 viruses, specifically the H5N1 and H1N1 subtypes. CR6261 and F10 neutralized H1N1, H2N2, H5N1, H6N1, H6N2, H8N4, and H9N2 viruses in vitro [41,52]. The prophylactic administration of 10 mg/kg F10 by the i.p. route one hour prior to lethal challenge with the H1N1 or H5N1 viruses led to the survival of 80–100% of the challenged mice. Similarly, the therapeutic administration of 15 mg/kg of F10 by the i.p. route at 1, 2, or 3 days post infection (dpi) with H5N1 viruses led to the 80–100% protection of infected mice. Prophylactic administration of 5 mg/kg of CR6261 by the i.p. route prior to the H5N1 lethal challenge led to 100% survival of challenged mice, whereas a lesser dose of 2 mg/kg fully protected mice from the H1N1 lethal challenge. The therapeutic administration of 15 mg/kg of CR6261 to mice 3–4 dpi with a lethal dose of H1N1 or H5N1 viruses provided full protection. Following three passages of H5N1 in cell culture in the presence of F10, no in vitro neutralization-resistant variants of H5N1 viruses were selected, whereas resistant variants were generated after 10 passages in the presence of CR6261 with a single H111L mutation in HA2 (Table 2). In a separate study, by combining the viral passaging (~11–12 passages in the presence of F10) and high-throughput sequencing, F10 resistance mutations in both HA (HA1:S111G, N205V; HA2:N116S) and NA (E329K) were identified, which were further confirmed independently in the reverse genetics system (Table 2) [70]. None of these mutations are localized in the stem epitope. Instead, they are located in the region surrounding the fusion peptide (N116S) or HA1–HA2 interface (S111G), which altered the HA stability (or fusion pH), and, in the region distal to stem (N205V), which altered receptor specificity [70]. As sbnAbs are also known to perturb NA activity, the resistance mutation in NA (E329K) effectively restored the HA/NA functional balance either by itself or in epistasis with N205V mutation in HA [70].

Both CR6261 and F10 share a similar epitope, which is highly conserved in the stem region of the group 1 HA subtypes (Figure 2A,B). Epitope binding is mediated via the heavy chain using HCDR1–3 and framework region 3 (FR3). The epitope comprises residues of helix A and a few residues of HA1. HCDR1 makes important contacts mainly with helix A residues and a few residues in the hydrophobic groove at the junction between the helix A and HA1 towards the membrane proximal end. In addition, the FR3 mediates minor contacts with the upper region of helix A. The conserved hydrophobic tip of HCDR2 contacts the hydrophobic groove in the membrane’s proximal end, whereas the base of HCDR3 contacts the lower part of helix A.

A few differences between group 1 and group 2 HAs restrict the epitope access of CR6261 and F10 to group 1 HAs. A highly conserved glycan (N38) on the HA1 of four out of six group 2 HAs, which is part of the epitope contacted by the heavy chain HCDR1, in addition to other group specific residues, prevent the cross-reactivity of CR6261 and F10 with group 2 HAs [26]. In addition, the angled orientation and positioning of HA2 W21 residue in group 2 viruses prevents contact with the HCDR2 loop [26].

#### 2.2.4. sbnAb 3.1

Using an H2 HA antigen, sbnAb 3.1 was isolated by the phage panning of variable regions derived from mature B cells (CD22+) of an individual previously vaccinated for influenza six times, but naïve for exposure to H2N2 viruses, which ceased to circulate after 1967. Like most V_H_1–69 germ line encoded sbnAbs, 3.1 potently neutralized group 1 viruses (H1, H2, H5, and H6). However, 3.1 displayed lower potency towards the H9 clade (H8, H9, and H12) and failed to neutralize the H1b clade (H11, H13, and H16) viruses in group 1. The sbnAb 3.1 has V_H_3–30 germline origin, like sbnAb FI6, and uses V_H_3–30, D3–9, J_H_4 gene segments for heavy chain, and V_κ_1–12/J_κ_4 gene segments for light chain. However, similar to other V_H_3–30- or V_H_1–69-sbnAbs, 3.1 exclusively uses its heavy chain to target the stem epitope.

The 3.1 epitope consists of residues from the N- and C-terminal regions of HA1 (38, 40 to 42, 289 to 293, and 318) and the N-terminal portion of HA2 (18 to 21, 38, 41, 42, 45, 49, 52, 53, 56), including helix A. The sbnAb 3.1 primarily uses HCDR1 and HCDR3 to make contacts with the hydrophobic groove. Furthermore, as observed with other sbnAbs (CR6261, FI6, C179) and peptide molecules (HB36 and F-HB80.4) specific to group 1 HA stem, the positioning and orientation of the W21 stem residue interacting with residue F100 in the HCDR3 tip of 3.1 is remarkably conserved [56]. Finally, the prophylactic administration of 10 mg/kg of 3.1 by the i.p. route prior to lethal challenge with the H1N1 virus led to the 100% survival of the challenged mice [56].

#### 2.2.5. PN-SIA49 and -SIA28

Both sbnAbs PN-SIA49 and -SIA28 were isolated from a 55-year-old vaccine recipient naive for 2009 H1N1pdm infection and without a clinical history of influenza virus in the past 10 years [57]. The germline usage of PN-SIA28 and PN-SIA49 comprised V_H_ genes, 3–30 and 3–23, respectively. Both sbnAbs displayed potent in vitro neutralization towards group 1 (H1N1, H1N1pdm, H2N2, H5N1) viruses except the H9N2 subtype [57]. The therapeutic administration of 10 mg/kg of PN-SIA49 led to 100% and 66.6% survival in the mice subjected to H1N1 and H5N1 lethal challenge, respectively [58]. PN-SIA49 targeted the conserved stem region in HA and competed the stem binding of C179. Although in vitro resistant variants of H1N1 viruses in the presence of PN-SIA49 were not identified, conserved stem residues critical for PN-SIA49 binding were identified by alanine-scanning mutagenesis (H34A, N338A, P338A on HA1; M360A, D19A, G20A, W21A, T41A, V55A, N56A, E60A on HA2) [58].

#### 2.2.6. FE43

sbnAb FE43 is one of the 20 bnAbs isolated through the limiting dilution cloning of EBV-immortalized memory B cells obtained from four donors, two weeks post vaccination, with 2007 trivalent inactivated seasonal influenza vaccine [55]. Ten out of the 20 bnAbs successfully competed with C179 against recombinant HA subtypes belonging to group 1 HAs, thus targeting a stem epitope similar to that of C179 [55]. This study demonstrated that heterosubtypic sbnAbs are elicited in individuals receiving seasonal influenza vaccination, although the extent of elicitation varies considerably between individuals and is generally well below the effective serum neutralizing concentration [55]. A major proportion of bnAbs including FE43 displayed V_H_1–69 germline usage (14 bnAbs), whereas germlines corresponding to V_H_3–23 (3 bnAbs), V_H_3–30, V_H_3–53, and V_H_4–39 were also identified in a monoclonal or polyclonal response from the same donor [55].

Most sbnAbs displayed the considerable breadth of viral neutralization towards group 1 subtypes including H1N1, H2N2, H5N1, H6N1, and H9N2, but failed to neutralize group 2 viruses [55]. FE43 efficiently neutralized H1, H5, H6 viruses, but failed to neutralize an avian H5N1 (A/VN/1203/04) and an H2N2 virus. Despite lacking the in vitro neutralization towards the H5N1 virus, FE43 displayed prophylactic efficacy and 100% protection when administered via the i.p. route at 25 mg/kg in mice prior to lethal challenge with avian H5N1, pointing to mechanisms (CDC, ADCP and ADCC) independent of neutralization [55]. In addition, FE43 also protected mice lethally challenged with H1N1, H5N1 (A/INA/5/05) and H6N1 (A/teal/Hong Kong/W312/97) viruses, with a relatively lower dose (2.5 mg/kg) conferring complete protection against H1N1 viruses [55]. Finally, the passive administration of FE43 also resulted in a significant reduction in the lung titers of H1N1-, H5N1-, H6N1-, and H9N2-infected mice.

#### 2.2.7. Small Proteins Targeting Group 1 HA Stem Domain

##### HB36 and HB80 Derivatives

The structural identification of sbnAb hot spot residues that contact hydrophobic pockets in the stem domain sparked the design of small proteins that bind the same pockets [59]. The design of these proteins involves the computational docking of individual hydrophobic residues (L, V, I, F, W, M, and Y) against stem epitopes in order to select hot spot residues that make energetically favorable interactions [59]. Then, these hot spot residues are placed on supporting scaffold proteins (~85–200 residues) that are shape-complementary to the stem region. The placement of hot spot residue side chains in appropriate conformation and configuration allows the design of peptides with optimized binding. Two such designs, namely HB36 and HB80 were identified that bound H1N1 (A/South Carolina/1/1918) HA with apparent moderate (K_d_ ~200nM) affinity and weak (K_d_ > 5000nM) affinity, respectively [59]. Variants of HB36 and HB80 with enhanced affinity were selected from a PCR mutant library and further optimized to produce HB36.4 (K_d_ ~4nM) and HB80.3 (K_d_ ~3nM), respectively [59,60].

A flag-tagged mini-protein variant of HB80.3 was further optimized to produce a high affinity binder, F-HB80.4, that bound to all group 1 HA (H1N1, H2N2, H5N1, H6N2, H9N2, H12N5, and H13N6) subtypes with low nanomolar affinity and neutralized H1N1 viruses [59,60]. Notably, F-HB80.4 also bound to H12 HA in contrast to HB80.3 and CR6261. Another high affinity variant of HB36, HB36.6, also bound to all H1 HAs with low nanomolar affinity and neutralized H1N1 viruses with potencies comparable to FI6v3 [60]. While mini-proteins lack the avidity advantage of bivalent antibodies, they may have a steric advantage in accessing the HA stem due to their smaller size compared to antibodies. The prophylactic i.n. administration of HB36.6 (6 mg/kg) two hours prior to lethal challenge with the H1N1pdm09 virus resulted in the 100% survival of mice with no weight loss [63]. A dose as low as 0.1 mg/kg also resulted in 100% survival, although the mice experienced transient weight loss [63]. This survival rate extended to other H1N1 (A/PR/8/34) and H5N1 (MN81) viruses as well, at a tested dose of 3 mg/kg [63]. Therapeutic i.n. administration of HB36.6 (3 mg/kg) on the day of H1N1pdm09 lethal challenge or daily until 4 dpi completely protected the mice. A dose-dependent increase in the survival rate was observed when HB36.6 was administered and 100% survival was observed when HB36.6 was administered with Oseltamivir [63]. Furthermore, the prophylactic or therapeutic administration of HB36.6 dampened lung viral replication and pulmonary immune pathology in lethally challenged mice [63].

##### HB1.6928.2.3

A massively parallel approach involving 22,600 mini-proteins with different backbone scaffolds of 37–43 residues was used to screen for HA binding [61]. Binders with a very high affinity (K_d_ < 10 nM), stability (T_m_ > 95 °C), and trypsin resistance were identified. Among them, one mini-protein HB1.6928.2.3 displayed potent neutralizing activity towards H1N1 (PR8) and H1N1pdm09 (CA09) viruses with a half-maximal effective concentration (EC_50_) value for CA09 more than 100-fold lower than sbnAb FI6v3 and HB36.6 [61]. Furthermore, the prophylactic administration of HB1.6928.2.3 (0.03 mg/kg) one day prior to the lethal challenge with H1N1pdm09 resulted in 100% survival. This was 100-fold lower than the dose of F16-v3 required for 100% survival. A single dose (3 mg/kg) of HB1.6928.2.3 administered within 72 h post-lethal challenge also resulted in 100% survival and only minimal weight loss [61]. The mini-protein route of delivery had a substantial impact on the survival outcome with the i.n. and intravenous (i.v.) routes conferring superior and little protection, respectively [61]. It is important to note that small proteins lack Fc-dependent effector mechanisms, and therefore robust protection is solely mediated by virus neutralization [61,63]. These findings highlight that localizing mini-proteins at the site of viral replication is critical for virus neutralization by mini-proteins, unlike sbnAbs (e.g., FI6v3) that mediate complete protection even when administered systemically. More importantly, the repeated injections of mini-proteins induced no observed inflammatory response, diminishing concerns over potential immunogenic and therapeutic side effects [61,63]. Finally, a recent advancement has been the development of peptide inhibitors of fusion with improved affinity and potency towards the group 1 HA stem by the virtue of peptide cyclization and the addition of non-proteinogenic amino acids [62].

### 2.3. Group 2 HA Stem-Neutralizing Antibodies

#### 2.3.1. sbnAb CR8020

The sbnAb CR8020 was the first reported sbnAb to group 2 HA and was isolated through limiting the dilution cloning of H3 (A/Wisconsin/67/2005) HA binding to the memory cells obtained from the 2006–2007 trivalent inactivated influenza vaccine recipients [27]. CR8020 binds to H3, H4, H7, H10, H14 and H15 HAs and displays in vitro neutralization activity against H3N2, H7N3, H7N7, and H10N7 viruses. The prophylactic i.n. administration of 3 mg/kg of CR8020 led to the 100% survival of mice lethally challenged with mouse-adapted H3N2 or H7N7 [27]. No signs of weight loss and respiratory distress were noted. The therapeutic administration of 15 mg/kg of CR8020 at 2 dpi with H3N2 virus, and 3 dpi with H7N7 virus, led to complete protection, preventing mice mortality [27].

The CR8020 uses both heavy and light chains to target an accessible epitope at the base of HA that is substantially closer to the viral membrane compared to the epitope for the group 1 sbnAb CR6261 (Figure 2A,C) [27]. Highly conserved among group 2 viruses, the epitope spans the C-terminal region of the fusion peptide (15–19 residues) and an outermost edge of the β-sheet recognized by HCDR1/3 and HCDR3, respectively. Only two residues are shared between the epitopes of CR6261 and CR8020, hence CR8020 lacks reactivity with group 1 viruses. Key differences between the group 1 and 2 HA stem that contribute to the lack of reactivity include a bulkier Y34 residue in group 1 HA in place of Q/T in group 2 HA, as well as a conserved glycan (N21) in group 1 HA1 subunit that sterically clashes with HCDR1 [27]. Regarding the mechanism of neutralization, CR8020 binds to group 2 HA in either the HA0 precursor or HA mature form to prevent cleavage to HA1 and HA2 or the conformational changes necessary for fusion, respectively [27].

Following four passages in the presence of CR8020, two neutralization-resistant variants of the A/Hong Kong/1/1968 (H3N2) virus were generated that contained a mutation (D19N or G33E) close to the CR8020 epitope (Table 2) [27]. Both variants are rare in natural isolates. Whereas D19N both disrupts a salt bridge with V_L_ R53 and destabilizes HA, G33E inserts a large side chain in the antibody–antigen interface to contribute to resistance [27].

#### 2.3.2. sbnAb CR8043

sbnAb CR8043, like CR8020, was isolated by immortalization and the limiting dilution cloning of H3 (A/Wisconsin/67/2005) HA bound-memory cells obtained from the 2007–2008 seasonal influenza vaccine recipients [46]. CR8043 Fab displayed high affinity binding to H3 and H10 HA subtypes, compared to its significantly lower affinity for the remaining group 2 subtypes (H4, H7, H14, and H15). In line with its binding affinity, CR8043 effectively neutralized H3N2 and H10N7 viruses in vitro, whereas no neutralizing activity against H7N3 and H7N7 viruses was observed [46]. A subtype-specific difference (Q34T) in H7 HA2 contributes to its resistance to neutralization by CR8043.

The prophylactic i.n. administration of CR8043 (3 mg/kg) led to the 100% survival of the mice lethally challenged with mouse-adapted H3N2, and a relatively higher dose of 30 mg/kg led to 100% survival after the H7N7 lethal challenge [46]. Thus, it appears that neutralization-independent activities, such as CDC, ADCC, and ADCP, contribute to protection from H7N7 viruses. Similar to CR8020, CR8043 utilizes both heavy and light chains to target a highly conserved epitope in the base of the stem, which consists of the fusion peptide, HA2 β-sheet, and neighboring helix A [46]. Following multiple in vitro passages in the presence of CR8043, two neutralization resistant-variants of A/Hong Kong/1/1968 (H3N2) were generated that bore an R25M or Q/T34R mutation close to the CR8020 epitope [46]. Interestingly, the Q34R mutation also conferred resistance to CR8020, whereas R25M failed to do so [46]. In addition, resistant variants (D19N, G33E) identified in the presence of CR8020 remained sensitive to CR8043 although with reduced potency (Table 2) [46]. Despite highly similar epitope interactions, this difference in sensitivity is due to the different angles of approach to HA and a six-residue insertion in LCDR1 of CR8043 (V_K_4–01 germ-line gene vs. V_K_3–20 for CR8020) [46].

#### 2.3.3. sbnAb 042-100809-2F04

The sbnAb 042-100809-2F04 was derived from the memory cells of seasonal trivalent influenza vaccine recipients. The sbnAb 042-100809-2F04 displayed cross-reactivity to H7N9 (A/Shanghai/1/2013 and A/Anhui/1/2013) viruses and was derived from the V_H_3–23/V_L_4–1 germline gene, and efficiently neutralized group 2 (H3N2, H7N9) viruses in vitro. At 1.5 mg/kg and 15 mg/kg, sbnAb 042-100809-2F04 prevented mortality in mice before and after lethal infection with H7N9 viruses, respectively. Furthermore, delayed therapy at 3 dpi also successfully prevented mortality. The sbnAb 042-100809-2F04 virus escape and antibody resistance involved the accumulation of mutations in the head (G71E, G241D) and stem (R25K) regions (Table 2). The 042-100809-2F04 epitope overlaps with the CR8020 epitope, and therefore, the R25K stem mutation confers resistance to both sbnAbs.

#### 2.3.4. Llama Single-Domain Antibodies (sdAbs) Targeting HA Stem

sdAbs targeting the HA stem were isolated from llamas prime immunized with the 2009/2010 inactivated influenza vaccine and boosted with recombinant HAs (H7 and H2 subtypes) [65]. The combinatorial phage libraries derived from the immunized llama PBMCs were panned against H1, H3, and influenza B (Victoria and Yamagata lineages) HAs [65]. The three stem-directed sdAbs, SD36, SD38 and SD83, and one head targeting sdAb, SD84, were generated. SD36 displays potent neutralization activity towards group 2 (H3N2, H7N9, H7N7, and H10N7), but not group 1 (H1N1, H2N2, and H5N1) and not group 2 viruses bearing the D46N mutation in the HA2 subunit that disrupts a salt bridge interaction. SD84, on the other hand, displays a potent and more modest neutralization towards group 1 (H1, H2, and H5) and group 2 (H3, H7, and H10) viruses, respectively [65].

Both the SD36 and SD38 recognize the conserved epitopes that partially overlap with the stem epitopes of sbnAbs CR9114, CR6261, and FI6v3, which includes helix A and the conserved stem residues shared by group 1 and group 2 HAs. The HCDR2 and HCDR3 of SD36 contacts residues on helix A in HA1 (N291, T318) and an adjacent HA1 (R32) protomer, whereas HCDR1 and HCDR3 contact HA2 residues (D19 and G20). SD36 FR1 contacts the upper region of the stem on HA1. A glycosylated residue (N289) on group 1 HA1 may induce a steric clash for binding by SD36, whereas SD38 avoids this glycan. The SD83 epitope includes conserved residues in the fusion subdomain [65]. The SD83 HCDR2 and HCDR3 contact the epitope comprising HA1 residues (30–32, K45, D291, N301, and P305), helix A, and N-linked glycans (N301 and N330), whereas the SR84 epitope comprises a conserved region surrounding the RBS of the HA head [65]. To enhance the neutralization potency and breadth, several fusions of sdAbs were made, including SD38–SD36, SD83–SD84, and SD38–SD36–SD83–SD84 (Multi domain 2407 or MD2407). SD38–SD36 demonstrated higher potency and broader cross-reactivity compared to individual sdAbs, and neutralized both group 1 and group 2 viruses, including the H3 viruses bearing the D46N mutation [65]. Similarly, SD83–SD84 also demonstrated higher neutralization potency towards the influenza B viruses. A broadly neutralizing MD3606 (MD2407 fused to human IgG1 Fc) demonstrated a much greater breadth and potency than the individual sdAbs or CR9114 and neutralized both influenza A and B viruses.

The prophylactic i.v. administration of MD3606 at 1.7, 5 and 1 mg/kg led to the 100% survival of the mice lethally challenged with H1N1, group 2 (H3N2, H7N9), and influenza B viruses, respectively [65]. Likewise, the prophylactic i.n. administration at 5 billion and 1 billion genome copies of recombinant adeno-associated virus vector 9-encoding MD3606 fully protected mice subjected to lethal challenge with H1N1, influenza B, and H3N2 viruses, respectively. Furthermore, MD3606-induced protection involves both neutralization and ADCC [65].

### 2.4. Group 1 and 2 HA Stem-Neutralizing Antibodies

#### 2.4.1. sbnAbs CR9114 and 1.12

CR9114 was derived through combinatorial phage libraries constructed from the memory B cells (CD24+CD27+IgM+) of a seasonal influenza vaccine recipient [42]. CR9114 bears remarkable breadth of potency and neutralization compared to other characterized sbnAbs. CR9114 displayed in vitro binding to the HAs derived from group 1 (H1, H2, H5, H9, H12, H13 and H13), group 2 (H3, H4, H7, H10, H14 and H15) influenza A viruses, as well as both lineages of influenza B viruses [42]. However, CR9114 in vitro neutralization capacity was limited to group 1 and group 2 influenza A viruses [42]. The prophylactic administration of 1.7 and 5 mg/kg CR9114 one day prior to challenging the mice with the respective lethal doses of H1N1 and H3N2 viruses protected all the animals from mortality. Furthermore, in contrast to its in vitro microneutralization capacity, relatively higher CR9114 doses of 15 mg/kg and 5 mg/kg also completely protected the mice from lethal challenge with B/Florida/4/2006 (Yamagata) and B/Malaysia/2506/2004 (Victoria) viruses, respectively [42]. Protection mechanisms could be mediated by Fc-dependent effector mechanisms.

CR9114 is a V_H_1–69 germline sbnAb that uses HCDR1–3 and FR3 for epitope recognition in the conserved stem region of HA [42]. The CR9114 epitope is nearly identical to the epitope of CR6261 and F10 and displays a similar mode of recognition using the HCDR or FR3 loops with no light chain contacts (Figure 2A,D). However, unlike CR6261 or F10, minor differences in CR9114 binding allows it to bind to a similar epitope in all influenza A viruses. First, CR9114 accommodates the larger N49 side chain in group 2 HA2 (T49 in group 1) [42]. Second, polymorphic residues at position 111 of the HA2 subunit in group 2 (T/A) and influenza B (E) compared to group 1 HA2 (H) result in the slightly altered orientation of W21 that affects its ability to interact with the HCDR2 hot spot residue (F21) of CR6261 and F10 [42]. CR9114 overcomes this through HCDR2 plasticity and a different orientation of F54, which allows the accommodation of minor orientation differences of the W21 residue [42]. Third, the conserved N38 glycan occluding the epitope surface in group 2 viruses is displaced by CR9114, whereas the same glycan (N332) in the influenza B virus appeared more difficult to avoid. Nevertheless, EM reconstructions demonstrated that CR9114 also binds to a similar epitope in the stem of influenza B HA [42].

Like CR9114, 1.12 is a V_H_1–69 germline antibody derived from phage libraries constructed from a seasonal influenza vaccine recipient. The sbnAb 1.12 broadly neutralized all the tested group 1 (H1N1, H1N1pdm09, H2N2, H5N3, H6N1, H8N4, H9N7, H11N9, H12N5) and group 2 (H3N2, H4N6, H7N7, H10N7, H13N6, H14N5) viruses [66]. Furthermore, the prophylactic i.p. administration of 1.12 followed by H1N1 and H3N2 lethal challenge resulted in 80% (10 mg/kg) and 100% (3 mg/kg) survival in the challenged mice, respectively [66]. Although sbnAb 1.12 utilizes its heavy chain for stem binding, its structural basis of stem interaction has yet to be solved [66].

#### 2.4.2. sbnAb FI6

FI6 was selected through limiting the dilution of plasma cells derived from a donor vaccinated with seasonal influenza and subsequently infected with swine-origin influenza (H1N1pdm09). FI6 bound to HAs belonging to both group 1 (H1, H2, H5, H6, H8, and H9) and group 2 (H3, H4, H7, and H10) viruses in binding ELISAs and stained cell surface HAs belonging to remaining group 1 (H11, H12, H13, and H16) and group 2 (H14, and H15) viruses [28]. FI6 had potent in vitro neutralization activity towards group 1 and group 2 viruses. FI6 displayed V_H_3–30*18 and V_K_4–1*01 germ line usage with a relatively long HCDR3 (22 amino acids) and a greater level of somatic hypermutation in both the V_H_ and V_K_ genes [28]. However, the investigation of neutralization potencies of germ line and somatically-mutated versions of FI6 revealed only two mutations (R93S and F27D-S) within the V_K_ chain of the germ line version to be critical for the neutralization of group 2 viruses. An optimized variant of FI6 lacking unnecessary and harmful residues called FI6v3 was constructed and displayed comparable binding and in vitro neutralizing properties to those of FI6 [28]. The epitope recognized by FI6v3 is similar to other sbnAbs CR9114, CR6261, and F10 (Figure 2) [28]. However, the angle of approach is remarkably different with a larger area of contact, including helix A and spanning the fusion peptide of the neighboring monomer in both cleaved and uncleaved HA forms [28]. In addition, the long HCDR3 by itself contacts the hydrophobic groove between HA1 and helix A. An F100 residue at the tip of the flexible HCDR3 loop interacts with different orientations of W21 in both group 1 and 2 viruses. Like CR9114, FI6v3 is able to reorient the conserved glycan at N38 away from the HA surface in order to prevent a steric clash with group 2 HAs [28].

Prophylactic i.v. administration of FI6v3 (4 mg/kg) provided complete protection from lethal challenge with H1N1 and prevented weight loss due to H3N2 HK-x31 non-lethal challenge at lower doses (1 mg/kg). The therapeutic administration of FI6v3 (15 mg/mg) 1 or 2 days after lethal infection with H1N1 also afforded complete protection. Potential mechanisms of FI6v3-induced protection include the inhibition of HA0 cleavage, HA fusion, CDC, and ADCC [28].

#### 2.4.3. sbnAb 045-051310-2B06

sbnAb 045-051310-2B06 was derived from the memory B cells of an H1N1pdm09 vaccine recipient by screening for H3 and H7 HA binders [64]. It displays V_H_1–18/V_K_3–11 germline usage and effectively neutralized both group 1 (H1N1, H5N1) and group 2 (H3N2, H7N9) viruses. It also displayed prophylactic and therapeutic efficacy at 1.5 and 15 mg/kg, respectively, in a mouse model of H7N9 lethal challenge with a 100% survival rate [64]. Escape mutants generated in the presence of 045-051310-2B06 after eight passages displayed two mutations in the stem region that overlapped with the CR9114 epitope (V325I in HA1 and I45N in HA2 subunit) and one in the globular head region (G202E) (Table 2) [64]. H7N9 viruses bearing I45N stem mutation displayed a loss of antibody binding and neutralization along with a loss of viral fitness in vivo.

#### 2.4.4. sbnAb S6-B01

S6-B01 was derived from the memory cells of 2006/07 seasonal trivalent influenza vaccine recipient and displayed cross-reactivity to H7N9 (A/Shanghai/1/2013 and A/Anhui/1/2013) viruses. S6-B01 had V_H_1–18/V_L_3–20 germline gene usage [64]. S6-B01 efficiently neutralized tested H5N1 but not H1N1 viruses from group 1, as well as group 2 (H3N2, H7N1, H7N3, H7N7, H7N9) viruses. At 1.5 and 15 mg/kg, S6-B01 displayed prophylactic and therapeutic efficacy in the mice lethally challenged with H7N9 viruses [64]. Escape mutants generated in the presence of S6-B01 bore mutations in both head (A205E, G221E) and stem (I45T) regions, although the stem mutation by itself was unable to confer neutralization resistance (Table 2) [64]. Likewise, this stem mutation located in the CR9114 epitope had no effect on CR9114 neutralization, potentially highlighting the similar epitope binding patterns of CR9114 and S6-B01 [64].

#### 2.4.5. sbnAb 3E1

sbnAb 3E1 is one of the seven bnAbs (1C4, 1E1, 1F2, 1F4, 1G1, 3C4, and 3E1) isolated from memory B-cells from a 27 year old, H1N1pdm09 split-virion vaccine recipient. The sbnAb 3E1 targets the conserved HA stem region of group 1 and group 2 viruses [69]. All seven bnAbs displayed more potent neutralization activity towards H1N1pdm09 compared to group 1 (H1N1, H5N1, H5N6, and H9N2) and group 2 (H3N2, H7N1) viruses. The DNA sequences of four of the bnAbs 1F2, 1F4, 1E1, and 1G1 revealed V_H_3–23 and V_L_3–15 gene usage, whereas two the bnAbs corresponded to V_H_3–30/V_L_3–20 (1C4) and V_H_1–69/V_L_3–20 (3C4) usage [69]. The DNA sequence of 3E1 indicated its germ line usage of IGHV4-4x07 and IGKV1-5x03 genes with less somatic hypermutation [71].

sbnAb 3E1 recognizes the conserved stem region of HA using both heavy and light chains. The 3E1 paratope comprises three HCDR loops, small portions of the three LCDR loops and HFR2. The 3E1 epitope is comprised of the C-terminus of the fusion peptide (residues 16–21 of HA2), part of the F subdomain (residues 38–52 of HA2 and residues 18, 38 and 326 of HA1), and the C-terminus of the outermost β-strand preceding helix A. In the upper region of the epitope, the HCDR3 loop and the three LCDR loops mainly interact with the F subdomain via hydrophobic interactions, whereas the LCDR loops make extensive hydrophobic contacts with helix A. At the lower region of the epitope, the HCDR2 loop interacts with the C-terminus of the outermost β-strand preceding helix A. The distinct conformational epitope of 3E1 combines the major regions of the epitopes recognized by both group 1 HA sbnAbs (CR6261, FI6v3, CR9114, F10, Fab3.1 and C179) and group 2 HA sbnAbs (CR8020, CR8043) [71]. Similar to group 1 sbnAbs, 3E1 makes extensive hydrophobic contacts with residues of the F subdomain via large hydrophobic and aromatic residues, and similar to group 2 sbnAbs, 3E1 contacts residues of the fusion peptide and the outermost β-strand. Although in vitro selection for resistance to 3E1 has not been undertaken, subtype-specific differences that contribute to the loss of 3E1 binding have been identified. An I45F mutation of HA2 in H2 subtypes causes steric hindrance with the HCDR3 loop of 3E1, resulting in abolished binding [71]. Similarly, a D19N mutation in HA2 of H13 and H16 subtypes adopts a different side-chain conformation that causes steric hindrance resulting in a loss of binding. A H18Q mutation in the HA1 fusion peptide C-terminus in H9 subtypes resulted in a loss of interaction with 3E1. Finally, an H38N glycosylation site in group 2 viruses obscures the 3E1 epitope resulting in a loss of recognition [71].

The sbnAb 3E1 displayed potent in vitro neutralization activity against group 1 (H1N1pdm09, H1N1, H5N1, H5N6) viruses [9]. The prophylactic i.p. administration of 3 mg/kg of 3E1 fully protected the mice subjected to challenge with H1N1pdm09 or H5N6 viruses [58]. The therapeutic administration of 20 mg/kg of 3E1 until 1 dpi also led to the 100% survival in mice previously infected with H1N1pdm09, whereas the H5N6-infected mice can be treated until 3 dpi to achieve 100% survival [71].

#### 2.4.6. sbnAb 3I14

The sbnAb 3I14 was isolated by the limiting dilution cloning of H3 (A/Brisbane/10/07) specific human memory B cells (CD19^+^CD27^+^) obtained from healthy donors [67]. The germline and binding configuration of 3I14 is identical to sbnAbs 39.29 and FI6v3, using both heavy and light chains for accessing a stem epitope. The sbnAb 3I14 effectively bound to the cell surface HA belonging to group 1 (H1, H2, H5, H6, H8, H9, H11, H12 and H16) and group 2 (H3, H4, H7, H14 and H15) [67]. The sbnAb 3I14 effectively neutralized both group 1 (H1N1, H1N1pdm09, H5N1) and group 2 (H3N2, H7N1, H7N9) viruses. The sbnAb 3I14 effectively cross-competed with other sbnAbs: FI6v3, CR9114, 39.29, F10, and CR8020 in binding to immobilized H3 HA, but was slightly less effective in binding to H5 HA [67]. The prophylactic administration of 5 mg/kg 3I14 by the i.p. route fully protected the mice from mortality due to the H7N7–NL219 or H7N9–AH13 lethal challenge, although challenged mice displayed minimal body weight loss at 14-18 dpi [67]. At a much higher dose of 25 mg/kg, 3I14 conferred 80% and 60% protection against the H3N2–BR07 and H5N1–VN04 challenge, respectively, with the surviving mice showing the reversal of weight loss by the end of the experimental period [67]. Protection mechanisms included the prevention of HA0 cleavage and HA fusion, as well as ADCC [67].

## 3. Conclusions and Future Directions

The discovery of sbnAbs with multi-subtype or even pan-flu reactivity has guided new vaccine strategies for eliciting sbnAbs [72]. Two strategies have led the way thus far. The first strategy involves immunization with headless HA lacking the immunodominant HA1 head domain in order to focus the immune response towards the conserved stem domain in the prefusion configuration [72]. Headless HA immunogen preparations have included stem constructs in various forms including CV-1 cell-surface expressed [73], sub-viral particles [74], soluble insect and HEK293F cell-expressed [75,76], HIV-1 gp41 or helical leucine zipper trimerization domain stabilized [76,77], isoleucine zipper coiled-coil or β-rich globular foldon trimerization motif stabilized [78] and ferritin nanoparticles [77]. Antibody responses generated by headless HA immunogens displayed limited rates of success in in vitro neutralization and in vivo heterosubtypic lethal challenge experiments. This observed scenario could be due to the alteration of the conformation of critical stem neutralization epitopes in the absence of globular HA head or the exposure of epitopes that are normally inaccessible in the presence of the HA head [75]. Headless HA immunogens may therefore induce a large proportion of Abs that cross react with immunogens but not the whole HA trimer. Eliciting high titers of antibodies capable of reaching stem epitopes in the context of the whole HA trimer remains a challenge.

A second strategy involves the sequential immunization with chimeric HAs (cHAs) that bear conserved stem domains from H1 and H3 subtypes, but novel head domains of avian influenza subtypes to which humans are usually naïve [79]. Typically, naïve animals require three sequential immunizations (1 prime + 2 boosts) with cHAs in order to focus the immune response towards the subdominant stem domain and induce protective immunity [80,81,82]. This strategy has proven successful in providing the complete protection of mice and ferrets subjected to heterosubtypic lethal challenge and reduced H1N1pdm09 transmission in ferrets [83,84]. Since individuals with a previous history of influenza vaccination and/or infection already carry rare sbnAbs, partly due to the less frequent existence of sbnAb-specific memory B cells or plasmablasts, it was hypothesized that immunization with cHA once or twice might suffice for the clonal expansion of sbnAb-specific cell populations to induce high titers of sbnAbs [72]. Nevertheless, age-dependent differences in the sbnAb pre- and post-vaccination titers call for an additional prime/boost strategy in children less than 5 months old, who had the lowest titers compared to the middle-aged adults and the elderly [85]. This strategy is analogous to the induction of sbnAbs in humans exposed to the novel HA of the 2009 pandemic virus, in which sbnAbs induced against the conserved stem may have contributed to the disappearance of all pre-pandemic H1N1 strains in the exposed human populations [72]. In this manner, sbnAbs induced by cHAs will likely bind and neutralize the HA of both circulating and pandemic viruses, whereas the Abs against novel head domains may also bear an advantage should a pandemic virus with a matched HA arise in the future [72]. However, findings in initial clinical studies have raised questions about the ability of repeated cHA protein immunizations to elicit and maintain high sbnAb titers [86,87]. Studies using cHA in various platforms are being investigated.

While efforts to develop novel vaccines that focus immune responses to the conserved HA stem continue, sbnAbs are being developed as therapy. A potential advantage of sbnAbs relative to other antiviral therapies, such as oseltamivir, is a greater genetic barrier to resistance to sbnAbs. At least in vitro, the generation of stem resistance generally requires a high number of passages (>10), often leading to a fitness cost in the resistant viruses that display attenuated phenotypes in vitro and in vivo [39,70]. However, a recent study has demonstrated a lower genetic barrier to resistance for H3 subtypes compared to H1 subtypes, with a low fitness cost for the H3 subtype resistant viruses in vitro and in vivo [88]. The existence of naturally occurring stem resistance mutations (I45T, I45M, and I45F) in circulating strains highlights the potential of emergence of resistance to stem epitopes upon immunological pressure on this region [88]. In addition, an HA2 L89I change in the HA stem, which first appeared in circulating H1N1 strains in 2006 and became dominant by 2008 before the pandemic virus took over in 2009, conferred a reduced sensitivity to neutralization by stem antibodies in clinical sera samples. This change suggested a possible drift in a stem epitope due to immune pressure [89]. For reasons of potential resistance, a combination of therapeutic sbnAbs with different resistance profiles could offer a meaningful complement or an alternative to existing antiviral therapy. Furthermore, the information gained from the structural characterization of HA–sbnAb interactions aids the rational design of small molecules, peptides, and protein inhibitors that mimic sbnAb interaction and therefore inhibit fusion. Likewise, knowledge gained from the somatic hypermutation and affinity maturation of sbnAbs informs the directed evolution of sbnAbs or peptides to improve binding affinity and potency. In summary, sbnAbs suggest new ways to overcome the hurdles associated with strain-specific vaccines.

## Figures and Tables

**Table 1:** List of stem-directed broadly neutralizing antibodies (sbnAbs) and their properties.

Stem Ab	Origin	Mode of Isolation	In Vitro Neutralization Potency and Breadth	Germ Line IGHV	Reference
C179	Mice twice immunized with A/Okuda/57 (H2N2)	Limiting dilution cloning of mice hybridomas reacting with H1N1 and H2N2 subtypes	Group 1 (H1, H2, H5, H6, H9)	NA	[38,50,51]
4C2	Mice sequentially immunized with H9 HA (in Freund’s complete adjuvant) + H9 HA (in Freund’s incomplete adjuvant) + 2 boosts of H5 HA + final boost (H9 + H2) HA	Screening of spleen hybridomas reacting with H5, H2 and H9 subtypes	Group 1 (H1, H2, H5, H9)	NA	[39]
CR6261	Seasonal influenza vaccinated human memory B cells (CD24+CD27+IgM+)	Combinatorial phage libraries	Group 1	IGHV1–69	[26,52]
CR6323	Seasonal influenza vaccinated human memory B cells (CD24+CD27+IgM+)	Combinatorial phage libraries	Group 1	IGHV1–69	[52]
F10	NA	Combinatorial phage libraries panned against H5N1 (A/Vietnam/1203/04) ectodomain	Group 1 (H1, H2, H5, H6, H8, H9, H11, H13, H16)	IGHV1–69	[41]
09-3A01	2009/10 seasonal trivalent inactivated vaccine (TIV) recipients vaccinated with pandemic (H1N1) 2009 vaccine	Single cell PCR of V_H_ and V_K_ genes of plasmablasts (CD19^+^CD3^−^CD20^lo/−^CD27^high^ CD38^high^)	Group 1	IGHV4–39	[33]
09-2A06	2009/10 seasonal TIV recipients vaccinated with pandemic (H1N1) 2009 vaccine	Single cell PCR of V_H_ and V_K_ genes of plasmablasts (CD19^+^CD3^−^CD20^lo/−^CD27^high^ CD38^high^)	Group 1	IGHV1–69	[33]
A06	Convalescent patients of A/Turkey/65596/06 (H5N1) infection	Combinatorial phage libraries panned against A/Vietnam/1203/04 (H5N1)	Group 1 (H1, H5)	IGHV1–69	[53,54]
39.18	2009 seasonal influenza vaccine recipient PBMCs (CD38+IgG+)	Antigen-specific plasmablasts enriched upon xenogenic transplantation in SCID mice	Group 1 (H1)	IGHV1–69	[37]
FE43	2007-08 seasonal TIV recipients two weeks post vaccination	Limiting dilution cloning of EBV-transformed B (CD22^+^IgM^–^IgD^–^IgA^–^) cells that displayed reactivity to H5 pseudovirus and seasonal H1N1 virus	Group 1 (H1, H5, H6, H9)	IGHV1–69	[55]
FE53	2007-08 seasonal TIV recipients two weeks post vaccination	Limiting dilution cloning of EBV-transformed B (CD22^+^IgM^–^IgD^–^IgA^–^) cells that displayed reactivity to H5 pseudovirus and seasonal H1N1 virus	Group 1 (H1, H5, H9)	IGHV1–69	[55]
70-1F02, 70-5B03, 1009-3B05, 1009-3E06, and 1000-3D04	Survivors of 2009 pandemic	Single cell RT-PCR of V_H_/V_K_ genes of antibody secreting cells	Group 1	IGHV1–69 and IGHV3–30 (1000-3D04)	[35]
Mab3.1	B cells (CD22+) of donor RI-13 vaccinated 6 times against seasonal influenza	Combinatorial phage libraries panned against H2N2 (A/Japan/1957) HA	Group 1 (H1, H2, H5, and H6 but not H11, H13, and H16); lower potency towards H9	IGHV3–30	[56]
PN-SIA49/-SIA28	B cells of influenza vaccine recipient aged 55, with a negative clinical history of influenza virus in the past 10 years	Limiting dilution of EBV-transformed B cells	Group 1 (H1, H1N1pdm, H2, H5 viruses, except the H9N2 subtype)	IGHV3–23	[57,58]
HB36.4	Computational design of disembodied hotspot amino acid residues docked against the target surface in an energetically favored manner and shape-complementary scaffolds that anchor these residues	Yeast surface display as a fusion protein and screening with biotinylated SC1918/H1 (A/South Carolina/1/1918 (H1N1)) HA ectodomain.	Group 1 (H1, H2, H5, H6)	NA	[59]
F-HB36.5	Integration of single-site mutagenesis libraries and multiple-segment Illumina sequencing with hot-spot–based computational protein interface design	1. Single site mutagenesis library transformed yeast cells screened for binders by selection with Viet/2004/H5 HA and SC1918/H1 HA and deep sequenced before and after selection. 2.The enriched substitutions are pooled into a final library, and optimized high-affinity variants are selected or designed from this pool	Group 1 (H1, H2, H5, H6, H9, H13, H16)	NA	[60]
HB80.3	Computational design of disembodied hotspot amino acid residues docked against the target surface in an energetically favored manner and shape-complementary scaffolds that anchor these residues	Yeast surface display as a fusion protein and screening with biotinylated SC1918/H1 (A/South Carolina/1/1918 (H1N1)) HA ectodomain.	Group 1 (H1, H2, H5, H6, H13, H16)	NA	[59]
F-HB80.4	Integration of single-site mutagenesis libraries and multiple-segment Illumina sequencing with hot-spot–based computational protein interface design	1. Single site mutagenesis library transformed yeast cells screened for binders by selection with Viet/2004/H5 HA and SC1918/H1 HA and deep sequenced before and after selection. 2.The enriched substitutions are pooled in a final library, and optimized high-affinity variants are selected or designed from this pool	Group 1 (H1, H2, H5, H6, H12, H13, H16)	NA	[60]
HB1.6928.2.3	De novo computational design of 7276 protein binders of HA, testing by yeast selection and deep sequencing	Yeast surface display as a fusion protein and screening with H1N1pdm09 (CA09) HA.	Group 1 (H1N1pdm09 and PR8)	NA	[61]
P7	De novo peptide design via key interacting residues of CR9114 (HCDR2, HCDR3, and FR3) and FI6v3 (HCDR3)	In vitro peptide affinity maturation and rigidification by cyclization and incorporation of non-proteinogenic amino acids	Group 1 (H1 and H5)	NA	[62]
HB36.6	Optimization of HB36.5 for higher affinity by combinatorial library substitutions	1. HB36.5 Single site mutagenesis library transformed yeast cells screened for high affinity binders with H1N1 (A/South Carolina/1/1918) HA. 2. The enriched substitutions are pooled in a final library, to design an optimized high-affinity variant with 9 substitutions of HB36.5	Group 1 (H1, H2, H5, H6, H9, H13, H16)	NA	[63]
CR8043	2007–2008 seasonal influenza vaccine recipient memory B cells (CD19+CD27+IgM+)	Limiting dilution cloning of H3 HA-bound (A/Wisconsin/67/2005) memory B cells	Group 2 (H3 and H10 subtypes)	IGHV1–3	[46]
CR8020	2006–2007 seasonal influenza vaccine recipient memory B cells (CD19+CD27+IgM+IgD+)	Limiting dilution of immortalized memory cells (CD19+CD27+IgD+) screened for APC-labeled H3 HA binding	Group 2 (H3, H7, and H10 subtypes)	IGHV1–18	[27]
36.89	2009 seasonal influenza vaccine recipient PBMCs (CD38+IgG+)	Antigen-specific plasmablasts enriched upon xenogenic transplantation in SCID mice	Group 2 (H3)	IGHV1–18	[37]
042-100809-2F04	Seasonal TIV (H3N2; A/Uruguay/716/2007) recipient	H3-reactive memory B cells screened for H7N9 (A/Shanghai/1/2013 and A/Anhui/1/2013) binding in an Ab microarray	Group 2 (H3, H7)	IGHV3–23	[64]
SD36	Llamas immunized with the 2009/2010 trivalent virosome subunit influenza vaccine+H7+H2 HA	Combinatorial phage libraries panned against H1 (A/New Caledonia/20/99), H3 (A/Brisbane/10/07), B/Florida/4/06 (Yamagata lineage) and B/Brisbane/60/08 (Victoria lineage).	Group 2 (H3, H4, H7, and H10)	NA	[65]
CR9114	Seasonal influenza vaccine recipient memory B cells (CD24+CD27+IgM+)	Combinatorial phage libraries panned against A/Wisconsin/67/2005 (H3), A/Netherlands/219/03 (H7), B/Ohio/01/2005 (Victoria lineage), B/Florida/4/2006 (Yamagata lineage), B/Brisbane/60/2008 (Victoria lineage) and A/duck/Hong Kong/24/1976 (H4)	Influenza A (Group 1 and 2; H1N1 and H3N2, respectively, except H2N2)	IGHV1–69	[42]
SFV005-2G02	2009/10 seasonal TIV recipients vaccinated with pandemic (H1N1) 2009 vaccine	Single cell PCR of V_H_ and V_K_ genes of plasmablasts (CD19^+^CD3^−^CD20^lo/−^CD27^high^ CD38^high^)	Group 1 and 2	IGHV1–18	[33]
CT149	Convalescent patients of H1N1pdm09 infection	Limiting dilution cloning of H3 HA-bound (A/Wisconsin/67/2005) memory B cells	Group 1 (lesser H1N1pdm09, H5, H9) and Group 2 (H3, H7)	IGHV1–18	[32]
CT164	Convalescent patients of H1N1pdm09 infection	Limiting dilution cloning of H3 HA-bound (A/Wisconsin/67/2005) memory B cells	Group 1 (H5) and Group 2 (H3)	NA	[32]
CT166	Convalescent patients of H1N1pdm09 infection	Limiting dilution cloning of H3 HA-bound (A/Wisconsin/67/2005) memory B cells	Group 1 (H5) and Group 2 (H3)	NA	[32]
FI6v3	Seasonal influenza vaccinated or swine-origin influenza infected human plasma cells (CD138^+^)	Limiting dilution cloning and HA-binding ELISA	Group 1 (H1, H2, H5, H6, H8, H9, H11, H12, H13, and H16) and Group 2 (H3, H4, H7, H14, and H15)	IGHV3–30	[28]
1.12	PBMCs of donor (RI13) vaccinated 6 times against influenza A virus prior to 2009 H1N1 pandemic	Combinatorial phage libraries of CD22^+^ B cells panned for H2 (A/Japan/305/1957(H2N2)), H3 (A/Moscow/10/1999(H3N2)), and H7 (A/fowl plague/Bratislava/1979 (H7N7)) HA binding.	Group 1 (H1N1, H1N1pdm09, H2N2, H5N3, H6N1, H8N4, H9N7, H11N9, H12N5) and group 2 (H3N2, H4N6, H7N7, H10N7, H13N6, H14N5)	IGHV1–69	[66]
3I14	Healthy donor PBMCs	Limiting dilution cloning of tetramerized H3 HA-bound (A/Brisbane/10/07) memory B cells (CD19^+^CD27^+^)	Group 1 (H1, H5) and Group 2 (H3, H7)	IGHV3–30	[67]
39.29	2009 seasonal influenza vaccine recipient PBMCs (CD38+IgG+)	Antigen-specific plasmablasts enriched upon xenogenic transplantation in SCID mice	Group 1 (H1, H2, H5) and Group 2 (H3, H7)	IGHV3–30	[37,68]
81.39	2009 seasonal influenza vaccine recipient PBMCs (CD38+IgG+)	Antigen-specific plasmablasts enriched upon xenogenic transplantation in SCID mice	Group 1 (H1, H2, H5) and Group 2 (H3, H7)	IGHV3–30	[37]
045-051310-2B06	Pandemic H1N1 (A/California/04/2009) vaccinated individual	H3-reactive memory B cells screened to bind A/Shanghai/1/2013 (H7N9) and A/Anhui/1/2013 (H7N9) in an Ab microarray	Group 1 (H1, and H5) and Group 2 (H3, H7)	IGHV1–18	[64]
S6-B01	2006/07 Seasonal TIV (H3N2; A/Wisconsin/67/2005) recipient	H3-reactive memory B cells screened for H7N9 (A/Shanghai/1/2013 and A/Anhui/1/2013) binding in an Ab microarray	Group 2 (H3, H7) and binds Group 1 (H1, H5) HA	IGHV1–18	[64]
SD38	Llamas immunized with the trivalent virosome subunit 2009/2010 influenza vaccine + H7 + H2 HA	Combinatorial phage libraries panned against A/New Caledonia/20/99 (H1), A/Brisbane/10/07 (H3), B/Florida/4/06 (Yamagata lineage) and B/Brisbane/60/08 (Victoria lineage).	Group 1 (H1, H2, and H5) and Group 2 (H3, H7, and H10)	NA	[65]
SD83	Llamas immunized with the trivalent virosome subunit 2009/2010 influenza vaccine + H7 + H2 HA	Combinatorial phage libraries panned against A/New Caledonia/20/99 (H1), A/Brisbane/10/07 (H3), B/Florida/4/06 (Yamagata lineage) and B/Brisbane/60/08 (Victoria lineage).	Influenza B lineages	NA	[65]
MEDI8852	Seasonal influenza vaccine recipient memory B cells (CD22^+^)	Limiting dilution cloning of H1N1 HA-bound (A/Vietnam/2005 H5N1 and A/Netherlands/2003 H7N7) memory B cells	Group 1 (H1, H2, H5, H6, H9, H11, H12, H13, H16, H17, H18) and Group 2 (H3, H7, H10, H14, H15)	IGHV6-1	[45]
mAbs (1C4, 1E1, 1F2, 1F4, 1G1, 3C4, and 3E1)	A healthy 2009 pandemic influenza vaccine recipient aged 27y	CA09 HA-specific memory B cells (CD19^+^IgG^+^BCR^+^ cells)	Group 1 (H1, H5, H9) and Group 2 (H3, H7)	IGHV3–30 (1C4), IGHV3–23 (1E1, 1F2, 1F4, 1G1), and IGHV1–69 (3C4)	[69]
54.f.01	H5 (A/Indonesia/05/2005) DNA vaccine followed by boosting with inactivated H5N1 vaccine (VRC310 trial)	PBMCs sorted by recombinant H1 (A/New Caledonia/20/1999), H5 (A/Indonesia/05/2005) or H3 (A/Perth/16/2009) probes followed by single-cell sequencing of heavy and light-chain genes, gene cloning and screening	Group 1 (H1, H2, H5) and Group 2 (H3, H7)	IGHV6–1	[44]
56.a.09	H5 (A/Indonesia/05/2005) DNA vaccine followed by boosting with inactivated H5N1 vaccine (VRC310 trial)	PBMCs sorted by recombinant H1 (A/New Caledonia/20/1999), H5 (A/Indonesia/05/2005) or H3 (A/Perth/16/2009) probes followed by single-cell sequencing of heavy and light-chain genes, gene cloning and screening	Group 1 (H1, H5) and Group 2 (H3, H7)	IGHV6–1	[44]
31.b.09	H5 (A/Indonesia/05/2005) DNA vaccine followed by boosting with inactivated H5N1 vaccine (VRC310 trial)	PBMCs sorted by recombinant H1 (A/New Caledonia/20/1999), H5 (A/Indonesia/05/2005) or H3 (A/Perth/16/2009) probes followed by single-cell sequencing of heavy and light-chain genes, gene cloning and screening	Group 1 (H1, H5) and Group 2 (H3, H7)	IGHV1–18	[44]
16.a.26	H5 (A/Indonesia/05/2005) DNA vaccine followed by boosting with inactivated H5N1 vaccine (VRC310 trial)	PBMCs sorted by recombinant H1 (A/New Caledonia/20/1999), H5 (A/Indonesia/05/2005) or H3 (A/Perth/16/2009) probes followed by single-cell sequencing of heavy and light-chain genes, gene cloning and screening	Group 1 (H1, H5, H9) and Group 2 (H3, H7)	IGHV1–18	[44]
31.a.83	H5 (A/Indonesia/05/2005) DNA vaccine followed by boosting with inactivated H5N1 vaccine (VRC310 trial)	PBMCs sorted by recombinant H1 (A/New Caledonia/20/1999), H5 (A/Indonesia/05/2005) or H3 (A/Perth/16/2009) probes followed by single-cell sequencing of heavy and light-chain genes, gene cloning and screening	Group 1 (H1, H2, H5, H9) and Group 2 (H3, H7)	IGHV3–23	[44]
16.g.07	H5 (A/Indonesia/05/2005) DNA vaccine followed by boosting with inactivated H5N1 vaccine (VRC310 trial)	PBMCs sorted by recombinant H1 (A/New Caledonia/20/1999), H5 (A/Indonesia/05/2005) or H3 (A/Perth/16/2009) probes followed by single-cell sequencing of heavy and light-chain genes, gene cloning and screening	Group 1 (H1, H5, H9) and Group 2 (H3, H7)	IGHV1–18	[44]
S9-1-10/5-1	Recipients of one or two influenza (H5N1) vaccines, an inactivated, adjuvanted whole-virus vaccine to A/Egypt/N03072/2010 or A/Indonesia/05/2005	PBMCs were fused with SPYMEG cells and hybridoma supernatants were screening for binding with H1N1pdm09, H5N1, H3N2, and H7N7	Group 1 (H1, H5) and Group 2 (H7 except H3)	IGHV4–59	[29]
MD3606	MD2407, fused form of SD38–SD36–SD83–SD84 fused to human IgG1 Fc	Fusion of llama single domain antibodies	Influenza A (H1 to H12 and H14) and B viruses	NA	[65]

NA—not available or not applicable in the case of HB36.4, F-HB36.5, HB80.3, F-HB80.4, HB1.6928.2.3, P7, HB36.6, SD38, SD83 and MD3606. Abbreviations: IGHV: Immunoglobulin heavy chain variable; SCID: Severe combined immune deficiency; EBV: Epstein Barr Virus; HCDR: Heavy chain complementary determining region; FR: framework region.

**Table 2:** List of the mutations and the subtype-specific differences conferring sbnAb resistance.

Heading	Stem Resistance Mutations (Subunit; Resistant Variant Subtype)		
sbnAb	In Vitro Passaging	Subtype Differences	Reference
C179	H38S, T318K (HA1; H2N2), V52E (HA2; H2N2)	H111T (HA2; H2N2)	[38]
4C2	K55E (HA1) and V444L (HA2) (H1N1)	-	[39]
CR6261 and CR6323	H111L (HA2, H5N1)	L320P (HA1; H2N2), H38N/Q40T (HA2; H3N2), I45F (HA2; H2N2), V52L (HA2; H3N2), S54R (HA2; H3N2)	[52]
F10	S111G, N205V (HA1; H1N1) N116S (HA2; H1N1)	-	[70]
CR8043	R25M, Q34R/T, (HA2; H3N2)	Q34T (HA2; H3N2)	[46]
CR8020	D19N, G33E (HA2; H3N2)	-	[27]
39.29	Q42K (helix A), D46Y, D46G (HA2; H1N1pdm09)	-	[68]
042-100809-2F04	G71E, G241D (HA1; H7N9) R25K (HA2)	-	[64]
045-051310-2B06	G202E V325I (HA1; H7N9) and I45N (HA2)	-	[64]
S6-B01	A205E, G221E, (HA1; H7N9) and I45T (HA2)	-	[64]
